# Gd-based protein cage nanoparticles provide enhanced r1 relaxivity and detect experimental atherosclerosis

**DOI:** 10.1186/1532-429X-13-S1-P370

**Published:** 2011-02-02

**Authors:** Lars O Liepold, Masaki Uchida, Md Joynald Abedin, Shefah Qazi, Hisanori Kosuge, Toshiro Kitagawa, Michael V McConnell, Trevor Douglas

**Affiliations:** 1 Montana State University, Bozeman, MT, USA; 2 Stanford University, Stanford, CA, USA

## Objective

Develop a highly sensitive T1 contrast agent based on chemically attaching a multitude of chelated Gd molecules constrained within a protein cage structure.

## Background

A T1 nanoparticle contrast agent showing high r1 relaxivity is desired to provide more sensitive molecular/cellular imaging with reduced Gd dose, and may have more clinical utility than T2* (e.g., iron-based) approaches. Tethering multiple Gd-chelates to a supramolecular platform is a promising strategy to increase r1 relaxivity, as rotational correlation time of the Gd ions can become significantly larger which is highly favorable for efficient r1 relaxivity. Here we utilize a small heat shock protein cage (Hsp) with a 12nm exterior diameter and a 9nm interior cavity, as a platform to anchor Gd-DTPA.

## Methods

### 1) Material development and evaluation

**Figure 1:**
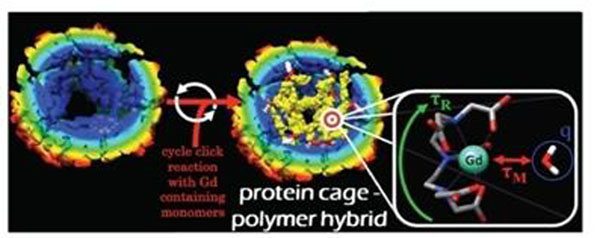
Illustration of Hsp-brach polymer with Gd.

Hsp was purified from an E. coli expression system. An azide-alkyne based click reaction is cycled to produce a branched polymer network in the interior of the protein cage (Fig 1). The polymer results in a stable network containing Gd-DTPA, as the azide-containing monomer has the Gd chelate attached prior to polymer generation.

### 2) In vivo imaging of vascular inflammation

FVB mice underwent left carotid ligation after 4 weeks of high-fat diet and diabetes induction by streptozotocin. Two weeks later, Hsp-Gd or Magnevist (Gd-DTPA) was intravenously injected at a dose of 20µmol Gd/kg (one-fifth the typical clinical dose). Mice were imaged on a whole-body 3T MRI scanner (Signa HDx, GE Healthcare) with a 50mT/m, 150T/m/s gradient system and a phased array mouse coil (RAPID MR International), using a T1-weighted fast spin echo sequence (TR/TE=400ms/15ms, slice thickness=1mm, FOV=3cm, matrix=256x256) before injection and 4h after injection.

## Results

**Figure 2:**
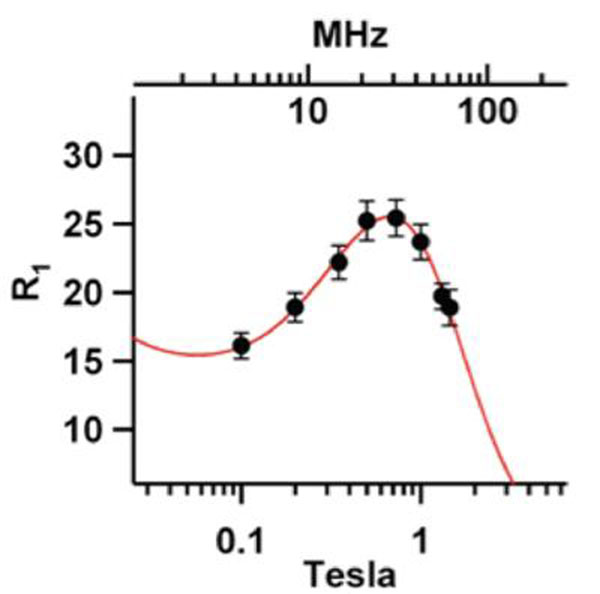
Graph of r1 measurement at varying field.

**Figure 3:**
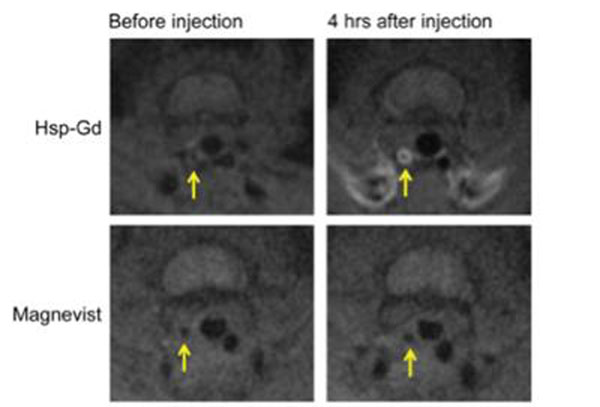
T1-weighted FSE MRI of ligated left carotids in mice before and after injection of Hsp-Gd (top) or Magnevist (bottom). The macrophage-rich left carotid lesion was clearly enhanced by Hsp-Gd, but not in the non-ligated right carotid artery or either carotid artery after Magnevist injection.

In vitro analysis of Hsp-Gd showed about 160 Gd-DTPA molecules per cage. At 0.73T, the ionic (per Gd) r1 value is 25mM^-1^sec^-1^ and the particle r1 value is 4,200mM^-1^sec^-1^ (Fig 2). At 3T, the ionic and particle r1 values are 9.7 mM^-1^sec^-1^ and 1600 mM^-1^sec^-1^, respectively. The ionic r1 value is nearly 3 times higher than that of Magnevist. The macrophage-rich left carotid lesion, but not the non-ligated right carotid, was clearly detected on T1-weighted MR imaging 4h after injection of Hsp-Gd, whereas the same lesion was hardly detected after Magnevist injection (Fig. 3).

## Conclusion

Gd can be effectively incorporated into polymer-incorporated protein cage nanoparticles providing high r1 relaxivity. Hsp-Gd allows positive contrast imaging of macrophage-rich carotid atherosclerosis with low Gd dosing. Thus, Gd-based protein cages are promising atherosclerosis imaging agents.

